# Baicalin inhibits the TGF-β1/p-Smad3 pathway to suppress epithelial-mesenchymal transition-induced metastasis in breast cancer

**DOI:** 10.18632/oncotarget.27677

**Published:** 2020-07-21

**Authors:** Ding-Kuo Liu, Hui-Feng Dong, Rui-Fen Liu, Xi-Long Xiao

**Affiliations:** ^1^ Department of Veterinary Medicine, China Agricultural University, Haidian District, Beijing, China; ^2^ S&E Burgeoning Biotechnology (Tianjin) Co., Ltd, Wangwenzhuang Industrial Park, Xiqing District, Tianjin, China; ^3^ Tianjin Key Laboratory of Microbial Preparation Enterprise for Feeding, Wangwenzhuang Industrial Park, Xiqing District, Tianjin, China; ^4^ Tianjin Chinese Veterinary Medicine Technology Engineering Center, Huayuan Industrial Park, Tianjin, China

**Keywords:** baicalin, TGF-β1, p-Smad3, epithelial-mesenchymal transition, breast cancer

## Abstract

TGF-β1 is an epithelial-mesenchymal transition (EMT)-inducing factor that is critical in tumor progression. However, whether the effect of TGF-β1 on breast cancer is through the EMT pathway remains to be determined, and drug development based on this mechanism needs to be improved. Results of this study showed that TGF-β1 dysregulation significantly correlated with the expression levels of EMT-associated markers and transcriptional factors. Exogenous expression of TGF-β1 promoted breast cancer cell metastasis and EMT progression. In addition, direct binding of baicalin to TGF-β1 caused its inactivation, which subsequently blocked signal transduction and inhibited breast cancer cell metastasis. *In vivo* experiment results further invalidated the inhibitory effect of baicalin on TGF-β1-induced tumor metastasis. These results suggest that baicalin, an active ingredient used in traditional Chinese medicine, exhibits a potential therapeutic effect on breast cancer metastasis by regulating TGF-β1-dependent EMT progression.

## INTRODUCTION

Breast cancer is the most common cancer among women, affecting over 1.5 million women each year. It causes the greatest number of cancer-related deaths. In 2015, 570,000 women died from breast cancer, approximately 15% of all cancer deaths among women. Surgery, chemotherapy, radiation therapy, hormonal therapy, targeted therapies, and complementary and holistic medicine are among the different therapies used for the treatment of breast cancer [1–8], but their many side effects, such as chemotherapy-induced pain and immune system inhibition, limit their applications.*Scutellaria baicalensis*, a heat-clearing and detoxifying herb used in Chinese medicine, is widely used in China because of its anti-inflammatory, antimicrobial, antioxidative, anti-cancer, and cardiomyocyte-protective pharmacological properties [9–13]. The flavonoid baicalin is isolated from dried roots of *S. baicalensis*. Studies indicated that baicalin could induce the apoptosis and inhibit the metastases, invasion, migration, and fibrosis of various cancers, including breast cancer, through multiple pathways [14–18]. High doses of baicalin cause certain toxicities and induce kidney injury and fibrosis.

Results of the present study showed that binding of baicalin to TGF-β1 inhibits TGF-β1, leading to the transcription blockage of TGF-β1 downstream genes. Downregulation of these genes renders breast cancer cells less mesenchymal. Moreover, *in vitro* and *in vivo* experiments revealed that baicalin inhibits tumor cell metastasis. This study provides a deep understanding of the role of TGF-β1 in breast cancer progression and a basis for the development of potential therapeutic substances in cancer based on TGF-β1-dependent EMT progression.

## RESULTS

### TGF-β1 is highly expressed in breast cancer and predicts poor prognosis

**Figure 1:**
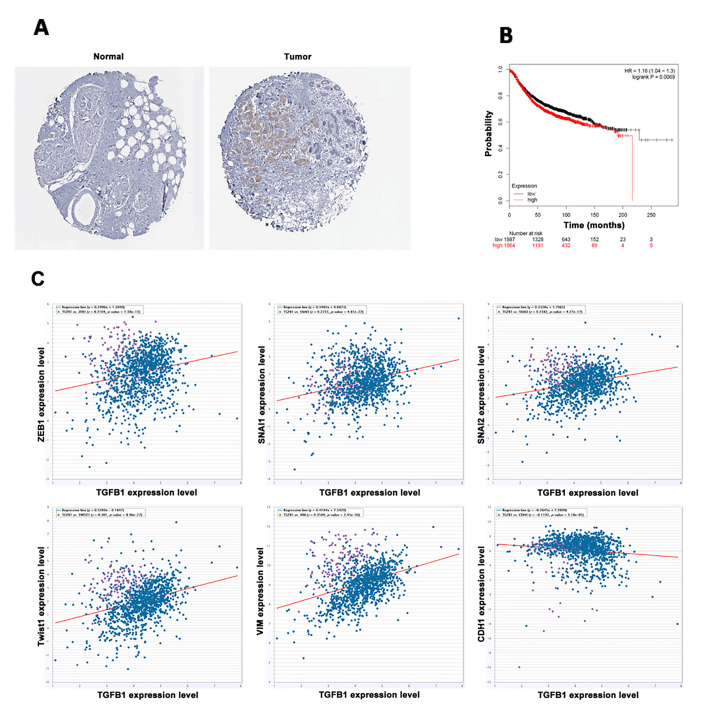
TGF-β1 was highly expressed in breast cancer and predicted poor prognosis. (**A**) The expression level of TGF-β1 was higher in breast cancer than in normal breast tissue. (**B**) High expression level of TGF-β1 predicted poor prognosis in breast cancer patients. (**C**) TGF-β1 was positively co-expressed with ZEB1, SNAI1, SNAI2, Twist1, and vimentin and negatively co-expressed with E-cadherin.

TGF-β1 is a potent EMT inductor with relatively high expression levels in various types of cancers. To verify the expression levels of TGF-β1 in breast cancer, we explored the HPA website (http://www.proteinatlas.org/) and obtained images of normal breast tissue and breast cancer tissue gained from immunohistochemical (IHC) assays. Results indicated that the expression level of TGF-β1 was higher in breast cancer than in normal breast tissue (Figure 1A). Subsequently, survival analysis was performed on the KM plotter website (http://kmplot.com/analysis/index.php?p=service). The survival curve revealed a negative correlation between the TGF-β1 expression and the lifetime of patients with breast cancer (Figure 1B). Ultimately, co-expression analysis was conducted between TGF-β1 and EMT markers, including ZEB1, SNAI1, SNAI2, Twist1, vimentin, and epithelial marker E-cadherin. The expression of TGF-β1 positively correlated with those of ZEB1, SNAI1, SNAI2, Twist1, and vimentin but negatively correlated with that of E-cadherin, indicating poor prognosis (Figure 1C).

### TGF-β1 promotes EMT

**Figure 2:**
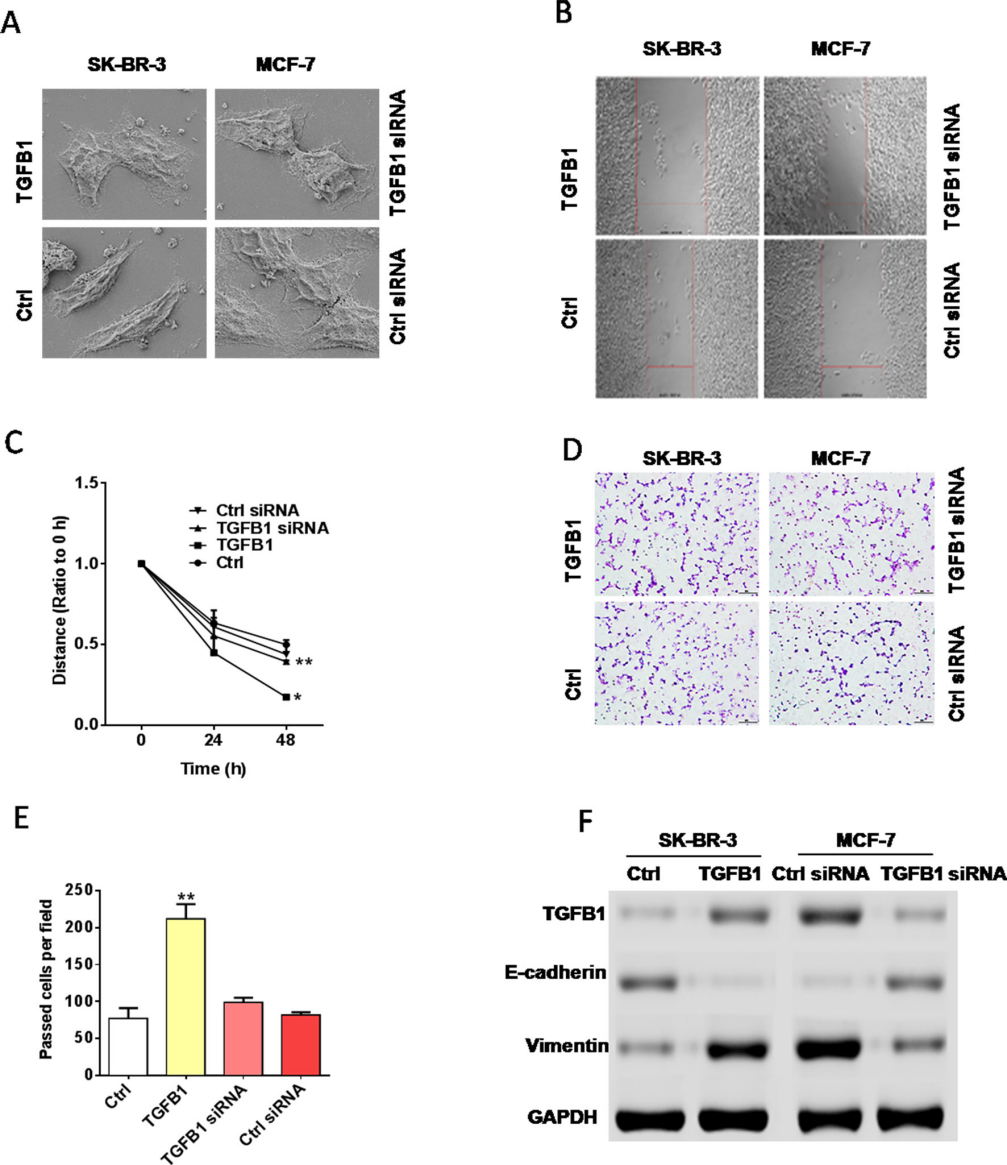
TGF-β1 promoted EMT in breast cancer. (**A**) SEM. TGF-β1 promoted the transition of two types of breast cancer cell lines from the epithelial phenotype to the mesenchymal phenotype. (**B**–**E**) Migration and invasion assays. TGF-β1 treatment could enhance the migration and invasion abilities of breast cancer cells compared with those in the control groups. (**F**) Western blot assay indicated that TGF-β1 treatment could upregulate the expression level of vimentin and downregulate that of E-cadherin, whereas the administration of TGF-β1 siRNA induced opposite effects.

To investigate the effect of TGF-β1 on EMT in breast cancer, scanning electron microscopy (SEM), western blot, and migration and invasion assays were conducted with cytokine TGF-β1 and corresponding siRNA. Breast cancer cell lines SK-BR-3 (with low TGF-β1 expression) and MCF-7 (with high TG1F-β1 expression) were used. SEM results showed that TGF-β1 promoted EMT in the SK-BR-3 cell line, whereas siRNA exerted the opposite effect on the MCF-7 cell line (Figure 2A). In migration and invasion assays, TGF-β1 strengthened the migration and invasion abilities of SK-BR-3 cells, whereas siRNA weakened these abilities in MCF-7 cells (Figure 2B–2E). Western blot assays implied that TGF-β1 could upregulate the expression levels of TGF-β1 and mesenchymal marker vimentin and downregulate the expression level of epithelial marker E-cadherin in SK-BR-3 cells. By contrast, siRNA downregulated the expression levels of TGF-β1 and mesenchymal marker vimentin and upregulated the expression level of epithelial marker E-cadherin in MCF-7 cells (Figure 2F). The assays above further confirmed the role of TGF-β1 in facilitating the progress of EMT.

### Baicalin inhibits EMT by downregulating the expression of TGF-β1 and p-Smad3

**Figure 3:**
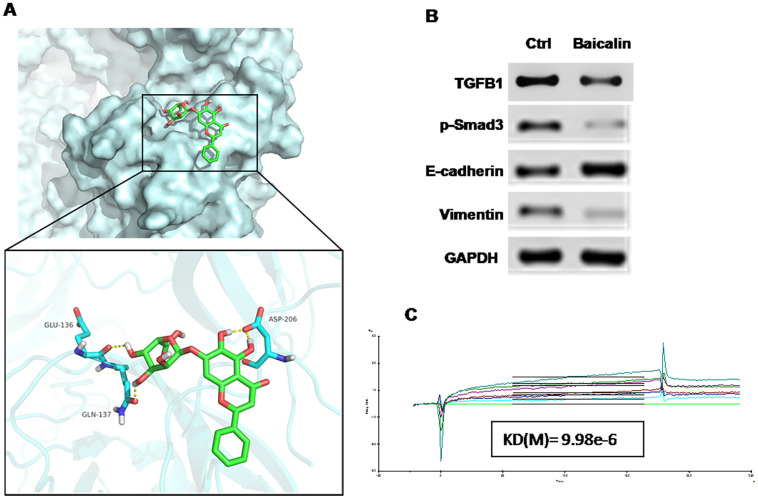
Baicalin suppressed EMT in breast cancer via targeting the TGF-β1/Snail1 pathway. (**A**) Molecular docking shows the strong interaction between TGF-β1 and baicalin. (**B**) Western blot assay performed with breast cancer cell line MCF-7 indicated that baicalin could upregulate the expression level of E-cadherin and downregulate those of TGF-β1, vimentin, and p-Smad3. (**C**) Biacore analysis revealed that baicalin could bind directly to TGF-β1.

Following the confirmation of the effect of TGF-β1 on EMT, we explored the bioactive molecules that inhibit this effect through the molecular docking method in the traditional Chinese medicine (TCM) database. Among the various TCMs, baicalin displayed a strong interaction with TGF-β1 (Figure 3A). Previous studies reported that baicalin may act as a potential drug that inhibits EMT in breast cancer. The results of Western blot showed that baicalin could apparently upregulate the expression level of E-cadherin but could downregulate those of TGF-β1, vimentin, and Smad3 compared with the control treatment (Figure 3B). Biacore analysis revealed that baicalin could bind directly to TGF-β1 (Figure 3C) and suppress EMT in breast cancer.

### Baicalin reverses the effect of TGF-β1 on EMT

**Figure 4:**
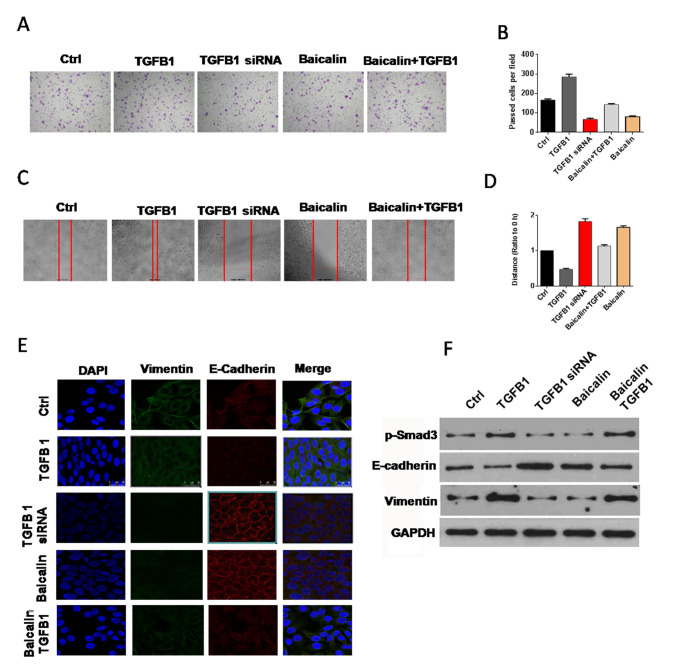
Baicalin counteracted the effect of TGF-β1 on EMT. (**A**–**D**) Baicalin reversed the facilitation effect of TGF-β1 on the migration and invasion abilities of breast cancer cells. (**E**) Immunofluorescence staining. Baicalin weakened the upregulation effect of TGF-β1 on vimentin expression and the downregulation effect of TGF-β1 on E-cadherin expression. (**F**) Western blot experiment demonstrated that TGF-β1 could downregulate the expression of E-cadherin and upregulate that of vimentin. However, the treatment of baicalin could counteract the effect of TGF-β1 on E-cadherin and vimentin expression. All of these assays were conducted with breast cancer cell line MCF-7.

Western blot, immunofluorescence staining, migration, and invasion assays were conducted to verify whether baicalin generates an anti-EMT effect by reversing the impact of TGF-β1. Compared with those of the cells in the control group, the migration and invasion abilities of the cells in baicalin-treated groups decreased significantly, whereas those of the cells in the baicalin/TGF-β1-treated groups decreased slightly (Figure 4A–4D). In the immunofluorescence staining assay, the E-cadherin immunofluorescence intensity of the cells in the baicalin-treated group was more enhanced compared with those of the cells in the control group, and vimentin immunofluorescence intensity receded sharply. In the baicalin/TGF-β1-treated group, the immunofluorescence intensity of E-cadherin enhanced slightly, whereas that of vimentin decreased mildly (Figure 4E). The result of Western blot indicated that the expression of E-cadherin was upregulated apparently, and the expression levels of TGF-β1 and p-Smad3 were downregulated in the baicalin-treated group. The expression levels of upregulated E-cadherin and downregulated TGF-β1 and p-Smad3 in the baicalin/TGF-β1-treated group were lower than those in the baicalin-treated group (Figure 4F).

### Baicalin suppresses metastasis of breast cancer *in vivo*

**Figure 5:**
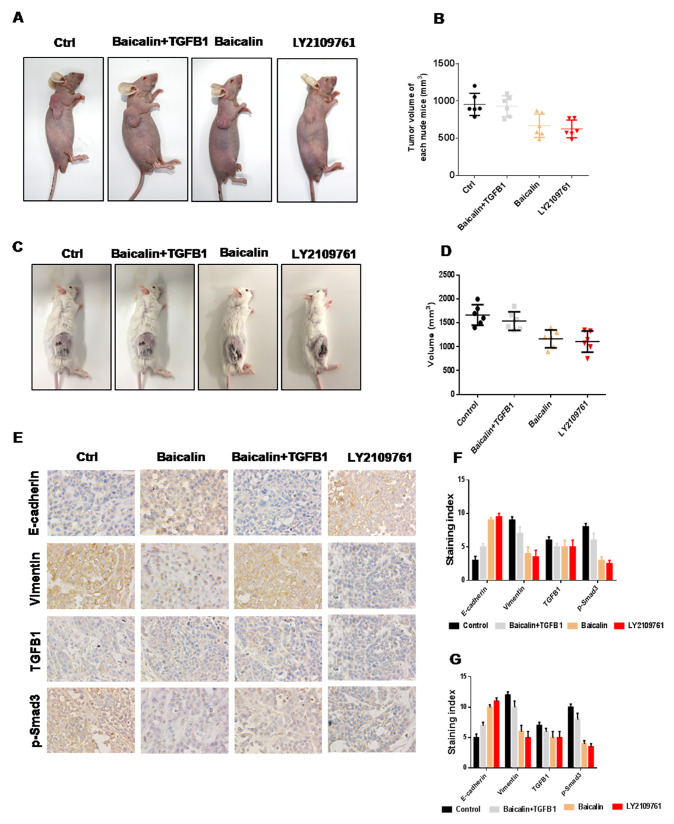
Baicalin suppressed the metastasis of breast cancer *in vivo*. (**A**–**D**) Baicalin inhibited tumor growth, whereas TGF-β1 promoted tumor growth in breast cancer. Baicalin upregulated the expression level of E-cadherin and downregulated those of TGF-β1, vimentin, and p-Smad3, whereas TGF-β1 induced opposite effects, in both BALB/c-null mice bearing MCF-7 cells (**E**–**F**) and BALB normal mice bearing 4T1 cells (**G**).

To investigate the effect of baicalin on breast cancer metastasis, an animal study was performed, followed by IHC analysis. In mice treated with baicalin alone, tumor volumes were smaller than those in the control group, whereas tumor volumes rebounded in the mice treated with baicalin and TGF-β1 (Figure 5A–5D). The results of IHC analysis indicated that the expression level of E-cadherin was upregulated, whereas those of TGF-β1, vimentin, and p-Smad3 were downregulated after treatment with baicalin in solid tumors. In the baicalin/TGF-β1-treated group, the expression level of E-cadherin was enhanced, whereas those of TGF-β1, vimentin, and Snail1 were decreased, however, the degrees of both were weaker than those in the baicalin-treated group (Figure 5E–5G).

## DISCUSSION

EMT [19–21] plays an important role in various pathological processes, including embryonic morphogenesis, wound healing, tissue fibrosis, and carcinoma progression. In these processes, EMT activation leads to the dissolution of epithelial cell–cell junctions and acquisition of motility and enables cell invasion. Upon activation of EMT, carcinoma cells lose their epithelial characteristics and acquire mesenchymal attributes, such as an elongated, fibroblast-like morphology, and an increased capacity for migration and invasion. In several types of carcinoma, forced induction of an EMT program in epithelial tumor cells substantially increases their capacity for tumor metastasis. In addition, EMT activates the resistance of tumor cells to many types of therapeutic agents. In breast cancer, the activation of EMT-inducing transcription factors, such as Slug, ZEB, Twist1, and TGF-β, induces EMT and promotes metastasis in breast cancer.

TGF-β [22–24] is a potent pleiotropic cytokine found in nearly all cell types and tissues and regulated by a negative regulatory feedback loop. Among the several isoforms of TGF-β, TGF-β1 is a predominant isoform in humans and plays pivotal roles in modulation of cellular growth, maturation, and differentiation; extracellular matrix formation; homeostasis; endothelial cell plasticity; immunoregulation; apoptosis; angiogenesis; and cancer progression. TGF-β1 signaling is transmitted into cells through specific membrane-binding receptors, and further signal transduction to the nucleus occurs with the participation of cytoplasmic proteins, including transcription factors and intracellular transmitters from the Smad family. Aside from their regulatory effect on transcription, Smads can also interact with other signaling pathways [e.g., nuclear factor-κB (NF-κB) pathways] under disease conditions.

Baicalin [25–29] is a flavonoid isolated from dried roots of *S. baicalensis*. This flavonoid can suppress tumor progression by inhibiting the migration, invasion, and metastasis and inducing the apoptosis and cell cycle arrest of tumor cells. These effects are obtained by regulating the p38MAPK and NF-κB pathways or upregulating P53 and bax. Meanwhile, baicalin could prevent normal mammary epithelial cells from undergoing the characteristic morphological changes into the fibroblastic phenotype.

In the present study, TGF-β1 was inactivated by baicalin, which inhibited TGF-β1-dependent EMT progression in breast cancer. This downregulation of TGF-β1 led to the repression of p-Smad3 and consequently reversed the inhibition of E-cadherin expression. Ultimately, the upregulation of E-cadherin resulted in the recovery of adhesive capacity among tumor cells and in the reduction of distal migration ability of these cells in breast cancer.

Overall, our results imply that baicalin inhibits breast cancer invasion by inhibiting EMT through suppressing the TGF-β1 signaling pathway. Our results provide a deep understanding of TGF-β1-dependent EMT progression and a new mechanism for the therapeutic application of baicalin in patients with breast cancer. Therefore, baicalin may serve as an effective alternative treatment for persistent carcinoma and a new candidate anti-metastatic drug.

## MATERIALS AND METHODS

### Cell culture

Human breast cell lines SK-BR-2 and MCF-7 were purchased from KeyGen Biotech (Nanjing, China) and cultured in Roswell Park Memorial Institute 1640 medium (Hyclone, USA) supplemented with 10% fetal bovine serum (FBS) (Hyclone, USA) at 37 °C under humidified conditions containing 5% CO_2_, trypsinized, and passaged every 2 days.

### Migration and invasion assays

For migration assays, cells were seeded in a 24-well plate at a density of 2 × 10^5^ cells/well. After 24 h, a wound was made in the center of the well, and baicalin (50 μM, purchased from ApexBio, TX, USA) or TGF-β1 (10 ng/mL, purchased from Affinity) was added and incubated for 48 h. Then, images were taken with a microscope.

Invasion assays were performed with a 24-well plate and 8 micron Matrigel Invasion Chambers. In brief, chambers were placed into the well of the plate, and 50 μL of Matrigel:medium (1:1) was added into the chambers. After solidification of the Matrigel, a 200 μL cell suspension without 10% FBS containing approximately 8 × 10^4^ cells and baicalin (50 μM) or TGF-β1 (10 ng/mL) was added into the upper chamber. Afterward, a 500 μL medium with 10% FBS acting as a chemo-attractant was added into the lower chamber, followed by incubation at 37 °C. After 24 h, the chambers were taken out, and the medium was abandoned. After washing thrice with 1× phosphate-buffered saline (PBS), the cells transferred through the filter membrane were fixed in 4% paraformaldehyde (precooled at 4 °C) and stained with crystal violet for 20 min at room temperature. At the end of the experiments, photographs were taken with a microscope.

### Immunofluorescence staining

Cells were seeded on the slide inside the well of the 24-well plate. After 20 h, the cells were added with baicalin (50 μM), TGF-β1 (10 ng/mL), or siRNA (100 nM) and then incubated at 37 °C for 24 h. They were washed thrice with 1× PBS, fixed in 4% paraformaldehyde (precooled at 4 °C) for 20 min, and then blocked with 5% bovine serum albumin (BSA) containing 0.1% TritonX-100 for 30 min at room temperature. Subsequently, the cells were incubated with primary antibodies diluted in 5% BSA. After washing thrice with 1× PBS, the cells were incubated with fluorescently conjugated secondary antibodies diluted in 5% BSA for about 50 min at room temperature under conditions protected from light. After four washes with 1× PBS, the cells were sealed with the mountant (containing DAPI), and images were taken with a laser-scanning confocal microscope (Nikon, Japan).

### SEM

Cells were seeded on climbing films and treated with TGF-β1 (10 ng/mL) or siRNA (100 nM) for 24 h. The cells were fixed and dehydrated in acetone/isoamyl acetate (1:1) and then dried with a gradient concentration of acetonitrile. After coating with gold, images were taken with a scanning electron microscope (LEO 1530 VP, Germany).

### Western blot

Cells were treated with baicalin (50 μM), TGF-β1 (10 ng/mL), or siRNA (100 nM) for 24 h. Proteins were extracted and quantified, and then whole-cell lysates were subjected to 10% SDS-PAGE, transferred onto PVDF membranes (Millipore, MA, USA), and then blocked with 5% nonfat milk. Subsequently, the membranes were incubated with primary antibodies, including E-cadherin, vimentin, TGF-β1, GAPDH, and p-Smad-3 (purchased from Affinity), and then diluted (dilution ratio: 1:1000) in blocking solution overnight at 4 °C. They were incubated with horseradish peroxidase-conjugated secondary antibody (1:5000) for 1 h at room temperature. Finally, the target protein bands were detected with Ultra-sensitive ECL luminescence solution and imaged with a Western Blot Imaging System (Clinx Science Instruments, Shanghai, China).

### Biacore analysis

SPR (surface plasmon resonance) experiments were performed using a Biacore 3000 instrument (GE Healthcare, NJ, USA). TGF-β1 was purified from the MCF-7 (TGF-β1 or mutated TGF-β1 was overexpressed) cell line using antibody-containing immunomagnetic beads. TGF-β1 was immobilized on CM5 sensor chips using the BiacoreAmini Coupling Kit in accordance with the manufacturer’s instructions. Baicalin was diluted in running buffer and then injected into TGF-β1-immobilized CM5 sensor chips at concentrations of 0, 1.5625, 3.125, 6.25, 12.5, 25, and 50 μM or 0, 20, 40, 80, 160, and 320 μM. The surface of the control chip was prepared in the same manner and used for data correction. Data analysis was performed using the BIA evaluation software.

### siRNA transfection

SK-BR-2 cells were transfected with TGF-β1siRNA using Lipofectamine RNAiMAX in accordance with the manufacturer’s instructions (Invitrogen, CA, USA). For RNA/Lipofectamine complex formation, siRNA was used at a working concentration of 100 nM, incubated for 15 min, added to the cells, and then incubated for 24 h. Then, the cells were harvested for Western blot assay or used for the migration and invasion assays and immunofluorescence staining.

### Murine xenograft model

Six-week-old female BALB/c-null and normal mice were used to test the effect of baicalin on breast cancer. Animal experiments were performed in accordance with the guidelines of the National Institutes of Health Animal Use. All experimental protocols were approved by the Institutional Animal Care and Use Committee at Tianjin International Joint Academy of Biomedicine. A total of 1 × 10^7^ cells (MCF-7 or 4T1) were injected subcutaneously to nude or normal mice. When tumor volume reached approximately 50 mm^3^, the mice were treated with baicalin (100 mg/kg) or TGF-β1 inhibitor TGLY2109761 (50 mg/kg purchased from Selleck, TX, USA) every day for 2 weeks. The mice were then monitored in 3-day intervals for tumor appearance. Tumor size was calculated using the following equation: tumor size = D^2^ L/2 (D = width, L = length). All mice were euthanized after 6 weeks of treatment.

### Immunohistochemical analysis

Tumor tissues from mice were fixed in 4% paraformaldehyde, embedded in paraffin, and then sectioned into 4 μm samples. Tissues were deparaffinized with xylene and then dehydrated with ethanol in decreasing concentrations. Endogenous peroxidase activity was blocked with 3% hydrogen peroxide, and the microwave antigen repair technique was utilized to retrieve antigens. After blocking, the histologic sections were incubated overnight at 4 °C with primary antibodies, including E-cadherin, vimentin, TGF-β1, and p-Smad-3 (dilution ratio: 1:200, Affinity). After washing with PBS, the tissue sections were incubated with biotinylated goat anti-mouse IgG antibody (Zhongshan Biology Technology Co., Ltd., Beijing, China) at 37 °C for 30 min. Subsequently, the sections were stained with 3,3′-diaminobenzidine/H_2_O_2_ and hematoxylin and eosin, cleared, and then mounted for observation and analysis.

### Statistical analysis

All results were presented as means ± standard deviation. Values were analyzed using Student’s *t*-test, ANOVA, and multivariate statistical analysis. The level of statistical significance was set at *P* < 0.05.

## References

[1] Abdulrahman Jnr GO Targeted therapies in the management of breast cancer. Gulf J Oncolog. 2015; 1:38–43. 26499829

[2] Almstedt K, Schmidt M Targeted Therapies Overcoming Endocrine Resistance in Hormone Receptor-Positive Breast Cancer. Breast Care (Basel). 2015; 10:168–72. 10.1159/000405017. 26557821 PMC4569254

[3] Chen H, Wu K, Wang M, Wang F, Zhang M, Zhang P A standard mastectomy should not be the only recommended breast surgical treatment for non-metastatic inflammatory breast cancer: A large population-based study in the Surveillance, Epidemiology, and End Results database 18. Breast. 2017; 35:48–54. 10.1016/j.breast.2017.06.002 28649032

[4] Geraci G, Fatica F, Cajozzo M, Anzalone AA, Modica G Surgical treatment of solitary sternal metastasis from breast cancer case report. Ann Ital Chir. 2016; 87:S2239253X16025196. 27585314

[5] Honig A, Rieger L, Sutterlin M, Wallwiener D, Dietl J, Solomayer EF State of the art of neoadjuvant chemotherapy in breast cancer: rationale, results and recent developments. Ger Med Sci. 2005; 3:Doc08. 19675725 PMC2703246

[6] Jordan VC, Brodie AM Development and evolution of therapies targeted to the estrogen receptor for the treatment and prevention of breast cancer. Steroids. 2007; 72:7–25. 10.1016/j.steroids.2006.10.009. 17169390 PMC2566956

[7] Mariani G New developments in the treatment of metastatic breast cancer: from chemotherapy to biological therapy. Ann Oncol. 2005; 16:ii191–94. 10.1093/annonc/mdi719. 15958455

[8] Weledji EP, Elong FA Primary surgical treatment of locally advanced breast cancer in low resource settings. Ann Med Surg (Lond). 2016; 12:5–7. 10.1016/j.amsu.2016.10.003. 27822368 PMC5090190

[9] Choi BY, Joo JC, Lee YK, Jang IS, Park SJ, Park YJ Anti-cancer effect of scutellaria baicalensis in combination with cisplatin in human ovarian cancer cell. BMC Complement Altern Med. 2017; 17:277. 10.1186/s12906-017-1776-2. 28545442 PMC5445329

[10] Chung CP, Park JB, Bae KH Pharmacological effects of methanolic extract from the root of scutellaria baicalensis and its flavonoids on human gingival fibroblast. Planta Med. 1995; 61:150–53. 10.1055/s-2006-958036. 7753922

[11] Kowalczyk E, Krzesiński P, Kura M, Niedworok J, Kowalski J, Błaszczyk J [Pharmacological effects of flavonoids from scutellaria baicalensis]. [Article in Polish]. Przegl Lek. 2006; 63:95–96. 16967717

[12] Kumagai T, Müller CI, Desmond JC, Imai Y, Heber D, Koeffler HP Scutellaria baicalensis, a herbal medicine: anti-proliferative and apoptotic activity against acute lymphocytic leukemia, lymphoma and myeloma cell lines. Leuk Res. 2007; 31:523–30. 10.1016/j.leukres.2006.08.019. 17007926

[13] Muluye RA, Bian Y, Alemu PN Anti-inflammatory and antimicrobial effects of heat-clearing chinese herbs: a current review. J Tradit Complement Med. 2014; 4:93–98. 10.4103/2225-4110.126635. 24860732 PMC4003708

[14] Chen J, Li Z, Chen AY, Ye X, Luo H, Rankin GO, Chen YC Inhibitory effect of baicalin and baicalein on ovarian cancer cells. Int J Mol Sci. 2013; 14:6012–25. 10.3390/ijms14036012. 23502466 PMC3634505

[15] Wang H, Li H, Chen F, Luo J, Gu J, Wang H, Wu H, Xu Y Baicalin extracted from huangqin (radix scutellariae baicalensis) induces apoptosis in gastric cancer cells by regulating B cell lymphoma (Bcl-2)/Bcl-2-associated X protein and activating caspase-3 and caspase-9. J Tradit Chin Med. 2017; 37:229–25. 10.1016/s0254-6272(17)30049-3. 29960296

[16] Wang XF, Zhou QM, Du J, Zhang H, Lu YY, Su SB Baicalin suppresses migration, invasion and metastasis of breast cancer via p38MAPK signaling pathway. Anticancer Agents Med Chem. 2013; 13:923–31. 10.2174/18715206113139990143. 23387975

[17] Won JH, Park KK, Chung WY Inhibitory effects of baicalein, baicalin and wogonin on osteolytic bone metastasis of breast cancer. Cancer Treat Rev. 2008; 34:S63–S64. 10.1016/j.ctrv.2008.03.065.

[18] Yu Y, Pei M, Li L Baicalin induces apoptosis in hepatic cancer cells *in vitro* and suppresses tumor growth *in vivo* . Int J Clin Exp Med. 2015; 8:8958–67. 26309548 PMC4538000

[19] Acloque H, Adams MS, Fishwick K, Bronner-Fraser M, Nieto MA Epithelial-mesenchymal transitions: the importance of changing cell state in development and disease. J Clin Invest. 2009; 119:1438–49. 10.1172/JCI38019. 19487820 PMC2689100

[20] Nieto MA Epithelial plasticity: a common theme in embryonic and cancer cells. Science. 2013; 342:1234850. 10.1126/science.1234850. 24202173

[21] Thiery JP, Acloque H, Huang RY, Nieto MA Epithelial-mesenchymal transitions in development and disease. Cell. 2009; 139:871–90. 10.1016/j.cell.2009.11.007. 19945376

[22] Kang JS, Liu C, Derynck R New regulatory mechanisms of TGF-beta receptor function. Trends Cell Biol. 2009; 19:385–94. 10.1016/j.tcb.2009.05.008. 19648010

[23] Massagué J TGFbeta in cancer. Cell. 2008; 134:215–30. 10.1016/j.cell.2008.07.001. 18662538 PMC3512574

[24] Moustakas A, Heldin CH The regulation of TGFbeta signal transduction. Development. 2009; 136:3699–714. 10.1242/dev.030338. 19855013

[25] Chung H, Choi HS, Seo EK, Kang DH, Oh ES Baicalin and baicalein inhibit transforming growth factor-β1-mediated epithelial-mesenchymal transition in human breast epithelial cells. Biochem Biophys Res Commun. 2015; 458:707–13. 10.1016/j.bbrc.2015.02.032. 25686495

[26] Franek KJ, Zhou Z, Zhang WD, Chen WY *In vitro* studies of baicalin alone or in combination with salvia miltiorrhiza extract as a potential anti-cancer agent . Int J Oncol. 2005; 26:217–24. 10.3892/ijo.26.1.217. 15586243

[27] Ikezoe T, Chen SS, Heber D, Taguchi H, Koeffler HP Baicalin is a major component of PC-SPES which inhibits the proliferation of human cancer cells via apoptosis and cell cycle arrest. Prostate. 2001; 49:285–92. 10.1002/pros.10024. 11746275

[28] Wang N, Tang LJ, Zhu GQ, Peng DY, Wang L, Sun FN, Li QL Apoptosis induced by baicalin involving up-regulation of P53 and bax in MCF-7 cells. J Asian Nat Prod Res. 2008; 10:1129–35. 10.1080/10286020802410664. 19031258

[29] Huang T, Liu Y, Zhang C Pharmacokinetics and Bioavailability Enhancement of Baicalin: A Review. Eur J Drug Metab Pharmacokinet. 2019; 44:159–168. 10.1007/s13318-018-0509-3. 30209794

